# The PCT to Albumin Ratio Predicts Mortality in Patients With Acute Kidney Injury Caused by Abdominal Infection-Evoked Sepsis

**DOI:** 10.3389/fnut.2021.584461

**Published:** 2021-06-01

**Authors:** Lijuan Chen, Xiaoli Wu, Haiyan Qin, Hongchao Zhu

**Affiliations:** ^1^Department of Pharmacy, The Affiliated Huaian No. 1 People's Hospital of Nanjing Medical University, Huaian, China; ^2^Department of Nuclear Medicine, The Affiliated Huaian No. 1 People's Hospital of Nanjing Medical University, Huaian, China

**Keywords:** procalcitonin, C-reactive protein, albumin, acute kidney injury, intra-abdominal infection

## Abstract

**Purpose:** Considerable evidence suggests that inflammation and malnutrition are common in patients with acute kidney injury (AKI) and correlated with mortality of various diseases. Despite this, few studies have reported the underlying predictive effects of inflammatory and nutritional markers in combination on the mortality of AKI patients. Herein, we aimed to explore the values of PCT and CRP as well as the ratios of PCT/Alb and CRP/Alb in the poor prognosis of patients with sepsis-induced AKI.

**Patients and Methods:** A total of 171 patients with AKI, caused by abdominal infection-evoked sepsis, were retrospectively studied and divided into a survival group (107 cases) and a non-survival group (64 cases). Univariate analysis was used to compare the clinical data of the two groups. Multivariate logistic regression analysis was used to analyze the independent risk factors of poor prognosis in patients with sepsis-induced AKI. The ROC curve was used to evaluate the diagnostic value of PCT, CRP, PCT/Alb, and CRP/Alb in the poor prognosis of patients with sepsis-induced AKI.

**Results:** Univariate analysis revealed that the values of PCT, CRP, CRP/Alb, and PCT/Alb were significantly different between AKI survival and non-survival groups, and further analysis found that PCT and PCT/Alb were independent risk factors for poor prognosis in patients with sepsis-induced AKI after adjusting with age and gender. Of note, the predictive accuracy (0.864 vs. 0.807), specificity (83.2 vs. 69.2), and sensitivity (79.7 vs. 76.6) of PCT/Alb were all higher than that of the simple PCT.

**Conclusions:** The ratio of PCT to Alb is an independent risk factor possessing a robust and accurate risk assessment for the poor prognosis of patients with sepsis-induced AKI, and it offers the potential to improve the management of this type of disease and a lower resultant mortality.

## Introduction

Sepsis, a systemic inflammatory response syndrome caused by infection, is mainly characterized by excessive release of inflammatory mediators and cytokines, which subsequently result in life-threatening organ dysfunction, especially in the heart and kidney. Although the implementation of the Surviving Sepsis Campaign (SSC) guidelines for sepsis management has effectively reduced the incidence of sepsis, sepsis shock still accounts for 62% of overall shock cases, with hospital mortality > 40% (1–3). Of the intensive care unit (ICU) patients, intra-abdominal infection (IAI) is the primary cause of sepsis, with an overall mortality of 10.5% worldwide (4). Notably, in patients with sepsis, the renal microvascular system is sensitive to vasoconstrictor substances, which is often concomitant with blockage of renal blood flow and diminishing glomerular filtration rate, and these kinds of pathological changes in the kidney also contribute to the resultant development of sepsis-induced AKI (SAKI). Indeed, more than 45% of patients with sepsis suffered from AKI, and all displayed poor outcomes (5, 6). Hence, owing to the hazardous effects of kidney sepsis, discovering robust predictive markers of mortality risk for sepsis-induced shock or AKI is imperative and will be beneficial for the management of such complications and future therapeutic intervention.

It has been well-known that inflammation and malnutrition are ubiquity in AKI patients (7), but few studies have suggested that inflammation and malnutrition, individually or in combination, were associated with the prognosis of AKI patients (7, 8). Moreover, so far, there is no research about the combination of inflammation and malnutrition markers to predict mortality in patients with sepsis-induced AKI. The purpose of our study was to assess the correlation among inflammation markers Serum C reactive protein (CRP), procalcitonin (PCT), and nutritional marker albumin (Alb) and 90-day mortality in sepsis-induced AKI and to put more attention on the combined markers (CRP/Alb and PCT/Alb). Considering that previous studies always found the markers in combination outperforming either marker alone in terms of making predictions of patients' prognosis, we speculated CRP/Alb or PCT/Alb might be the valuable marker for predicting mortality in sepsis-induced AKI patients.

## Patients and Methods

### Clinical Definition

IAI was defined as an infection limited to a hollow viscus or extended into a sterile area of the abdomen, such as the peritoneal cavity, mesentery, retroperitoneum, and abdomen wall (9, 10). Sepsis met the clinical criteria for septic shock [The Third International Consensus Definitions for Sepsis and Septic Shock (Sepsis-3)] (1). Patients with AKI were enrolled in conformance with the criteria of the Kidney Disease Improving Global Outcomes (KDIGO) classification (11), and this was based on the serum creatinine increasing by ≥0.3 mg/dl (≥26.5 μmol/l) within 48 h or the serum creatinine level increasing ≥1.5 times over the baseline level within 7 days or cumulative 6 h urine output ≤0.5 ml/kg/h.

### Patients

**Figure 1:**
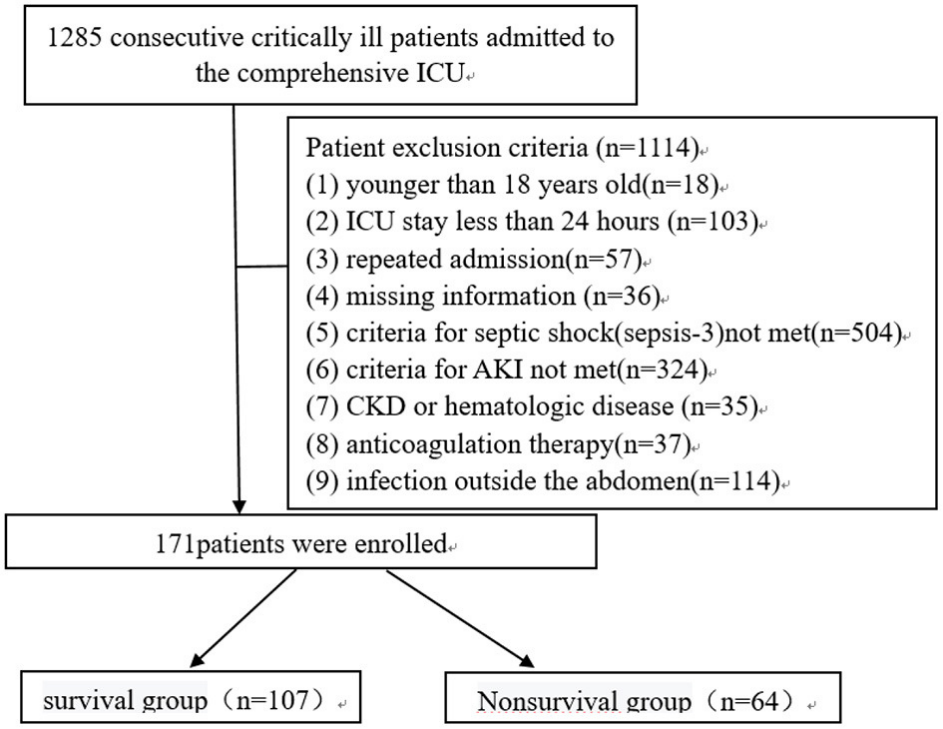
Flowchart of the studied patients.

This observational retrospective study was conducted in the comprehensive ICU of the Affiliated Huaian No. 1 People's Hospital of Nanjing Medical University. From January 1, 2016, to December 31, 2019, a total of 1,285 critically ill patients were admitted to the ICU. Among these admissions, 171 patients who met the criteria of septic shock-induced AKI [The Third International Consensus Definitions for Sepsis and Septic Shock (Sepsis-3)] were studied (Figure 1). The exclusion criteria were applied and are depicted in Figure 1. Based on their prognosis, they were divided into two groups: the survival group (107 cases) and the non-survival group (64 cases). The study was approved by the ethics committee of Huaian No. 1 People's Hospital (date: September 10, 2020; approval number: YX-P-2020-153-01).

### Data Collection

The data of patients were from electronic and paper medical records and recorded on electronic forms, including the following aspects: (1) demographic data: age, sex, weight, and body mass index (BMI); (2) admission status data: the source of IAI, comorbidities (hypertension and diabetes); and (3) laboratory data within 24 h of admission: serum procalcitonin (PCT), serum creatinine (SCr), serum albumin (Alb), white blood cell (WBC), neutrophil percentage (NEUT%), C-reactive protein (CRP), PCT/Alb ratio, CRP/Alb ratio, use of renal replacement therapy (CRRT), ventilation duration, and length of ICU stay.

## Statistical Analysis

Continuous variables were expressed as mean ± SD for normally distributed variables or median with interquartile range for non-normally distributed variables, and categorical variables were expressed as percentages. In univariate analysis, a student *t*-test or Mann-Whitney U-test was performed to compare continuous variables, and a Pearson chi test or Fisher exact test was used to compare the categorical variables. For multivariate logistic regression analysis, PCT and PCT/Alb were served as continuous variables, and we adjusted age (a continuous variable) and gender (a categorical variable), two confounding factors, to remove the influence of confounding factors and to achieve high predictive accuracy of PCT and PCT/Albumin (the two primary indicators) in mortality of sepsis-induced AKI patients. The ROC curve was applied to analyze and evaluate the diagnostic value of PCT, CRP, CRP/Alb, and PCT/Alb in the poor prognosis of patients (i.e., 90-day mortality) with sepsis-induced AKI. The power analysis (checking the sample size) and statistical analysis were performed using PASS 15.0 and SPSS 22.0 software packages, respectively (IBM, Chicago, Illinois, USA). The power analysis showed that the minimum number for our experimental design was forty-one, and our simple size met this demand. The value of *P* < 0.05 was considered statistically significant.

## Results

### Patient Characteristics and Etiologies of Abdominal Infection

As presented in Table 1, mortality for abdominal abscess (caused by small intestinal necrosis or perforation disease) was significantly higher in patients (32.81%, *P* < 0.01), while mortality for the ileocecal and appendiceal disease was relatively lower (3.12%, *P* < 0.05). However, in respect to other etiologies of intra-abdominal infection, the mortality among patients showed no differences (*P* > 0.05). Table 1 also displayed the demographic and clinical features, including age, gender, weight, BMI, comorbidities (hypertension and diabetes), length of ICU stay, ventilation duration, and CRRT; however, apart from weight and BMI, the remaining features displayed no differences between the survival group and the non-survival group (*P* > 0.05).

**Table 1:** Baseline characteristics of patients between survival group and non-survival groups and etiologies of intra-abdominal infection.

	**Survival group (*n* = 107)**	**Non-survival group (*n* = 64)**	***P-value***
Age (y) (mean ± SD)	75.40 ± 10.78	73.76 ± 10.69	0.663
Male sex, *n* (%)	55 (51.40)	34 (53.12)	0.718
Weight (kg)	65.49 ± 11.23	60.37 ± 10.56	0.039
BMI (kg/m^2^)	23.41 ± 2.76	22.04 ± 2.21	0.016
Hypertension, *n* (%)	64 (59.81)	25 (39.06)	0.120
Diabetes, *n* (%)	32 (29.90)	17 (26.56)	0.202
WBC (g/l) (mean ± SD)	18.18 ± 3.93	23.62 ± 17.22	0.328
NEUT% (mean ± SD)	92.52 ± 5.23	91.80 ± 8.61	0.802
Length of ICU stay (days), median (IQR)	10.5 (5.25–38.5)	7 (4–13)	0.864
Ventilation duration (days), median (IQR)	4.0 (0.75–34.75)	6 (3–11.5)	0.242
CRRT, *n* (%)	67 (62.62)	38 (59.37)	0.993
Gastroduodenal disease, *n* (%)	36 (33.64)	10 (15.62)	0.058
Biliary disease, *n* (%)	20 (18.69)	9 (14.06)	0.590
Colorectal disease, *n* (%)	24 (22.42)	13 (20.31)	0.838
Ileocecal and appendiceal disease, *n* (%)	16 (14.95)	2 (3.12)	0.040
Abdominal abscess, *n* (%)	4 (3.74)	21 (32.81)	0.003
Severe acute pancreatitis, *n* (%)	7 (6.5)	9 (14.06)	0.374

*Continuous variables were expressed as mean ± SD or median with interquartile range, and categorical variables were expressed as a percentage*.

*IQR, range of quartile; WBC, white blood cell; NEUT%, neutrophil percentage; CRRT, renal replacement therapy; ICU, intensive care unit*.

### Univariate Analysis of Selected Inflammation and Nutritional Indicators in Patients With Sepsis-Induced AKI

Table 2 depicts the inflammation and nutritional indicators obtained from the blood of involved patients within 24 h of admission, including CRP, PCT, and Alb. Compared with the survival group, the concentration of PCT [52.57 (19.16–100.00) mg/l vs. 8.74 (1.07–21.93) mg/l, *P* < 0.001] and CRP [224.38 (188.13–276.77) mg/l vs. 186.33 (136.33–235.85) mg/l, *P* = 0.008] significantly increased in the non-survival group. In contrast, Alb, a marker used to reflect the nutritional status, showed a slight (non-significant) downward trend in the non-survival group when compared to the survival group (22.15 ± 5.48 g/l vs. 24.41 ± 4.72 g/l). Most notably, we found that the ratio of PCT/Alb and CRP/Alb in the non-survival group were both remarkably higher than that of the survival group [for PCT/Alb: 2.43 (2.93) vs. 0.29 (0.49), *P* < 0.001; for CRP/Alb: 10.81 (6.65) vs. 5.84 (5.58.), *P* < 0.001].

**Table 2:** Univariate analysis of PCT, PCT/Alb, CRP, and CRP/Alb between survival and non-survival groups in patients with sepsis-induced AKI.

	**Survival group (*n* = 107)**	**Non-survival group (*n* = 64)**	***P*-value**
PCT (ng/ml), median (IQR)	8.74 (1.07–21.93)	52.57 (19.16–100.00)	0.000
CRP (mg/l), median (IQR)	186.33 (136.33–235.85)	224.38 (188.13–276.77)	0.008
Alb(g/l) (mean ± SD)	24.41 ± 4.72	22.15 ± 5.48	0.234
CRP/Alb, median (IQR)	5.84 (5.34–10.92)	10.81 (7.14–13.79)	0.001
PCT/Alb, median (IQR)	0.29 (0.04–0.53)	2.43 (0.85–3.79)	0.000

*IQR, range of quartile; Alb, Serum albumin; CRP, C-reactive protein; PCT, procalcitonin; CRP/Alb, the ratio of CRP to Alb; PCT/Alb, the ratio of PCT to Alb*.

### Multivariate Logistic Regression Analysis of Possible Predictors of Mortality in Sepsis-Induced AKI Patients

**Figure 2:**
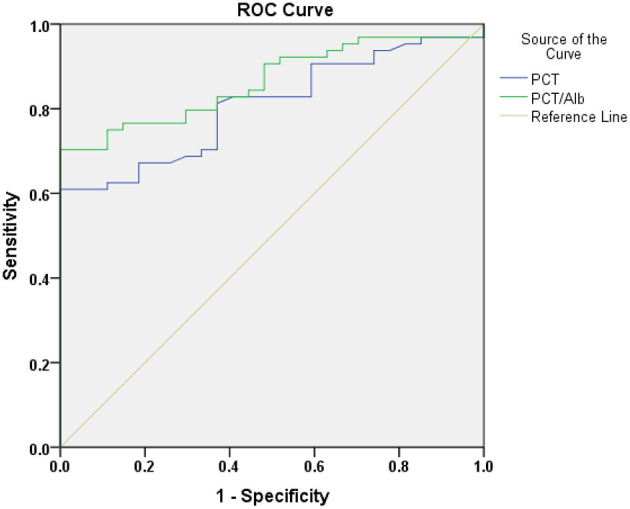
The predictive value of PCT and PCT/Alb in the poor prognosis of sepsis-induced AKI patients.

As shown in Table 3, after adjustment for age and gender, multivariate logistic regression analysis revealed that the odds ratio (OR) of PCT [OR, 1.060; 95% confidence interval (CI), 1.016–1.107] and PCT/Alb [OR, 2.372; 95% confidence interval (CI), 1.154–4.878] were >1 and that the *P*-values were all <0.05 (on the basis of *P* < 0.05, OR > 1 indicated the increase of risk and the higher the PCT and PCT/Alb value, the greater the mortality). Overall, both PCT and PCT/Alb were independent risk factors for poor prognosis in patients with sepsis-induced AKI. To further evaluate the diagnostic value of PCT and PCT/Alb in sepsis-induced AKI patients, the possible predictors were analyzed using the receiver operating characteristic (ROC) curve. As shown in Table 4 and Figure 2, the predictive accuracy (0.864 vs. 0.807), specificity (83.2 vs. 69.2), and sensitivity (79.7 vs. 76.6) of PCT/Alb were all higher than the simple PCT, indicating that PCT/Alb might be a robust risk assessment marker for the poor prognosis of patients with sepsis-induced AKI.

**Table 3:** Multivariate logistic regression analysis of predictive values of PCT, PCT/Alb, CRP, and CRP/Alb in patients with sepsis-induced AKI.

**Variable**	**Unadjusted**					**Adjusted**				
	**β**	**SE**	**Wald**	**OR (95% Cl)**	***P-*value**	**β**	**SE**	**Wald**	**OR (95% Cl)**	***P-*value**
PCT	0.059	0.022	7.081	1.060 (1.016–1.107)	0.008	0.065	0.027	5.889	1.067 (1.013–1.125)	0.015
PCT/Alb	0.864	0.368	5.517	2.372 (1.154–4.878)	0.019	0.979	0.459	4.544	2.662 (1.082–6.549)	0.033
CRP	0.014	0.011	1.624	1.014 (0.992–1.037)	0.203	0.023	0.013	3.120	1.023 (0.998–1.049)	0.077
CRP/Alb	0.229	0.183	1.563	1.257 (0.878–1.800)	0.211	0.152	0.202	0.563	1.164 (0.783–1.730)	0.453

*Alb, Serum albumin; CRP, C-reactive protein; PCT, procalcitonin; CRP/Alb, the ratio of CRP to Alb; PCT/Alb, the ratio of PCT to Alb*.

**Table 4:** PCT, PCT/Alb, CRP, and CRP/Alb were analyzed by the ROC curve.

**Variable**	**AUC**	**95% Cl**	**Sensitivity (%)**	**Specificity (%)**	**Youden index**	**Critical value**
PCT	0.807	0.740–0.875	76.6	69.2	0.458	18.455
PCT/Alb	0.864	0.807–0.922	79.7	83.2	0.629	0.6827

*Alb, Serum albumin; PCT, procalcitonin; PCT/Alb, the ratio of PCT to Alb*.

## Discussion

We retrospectively assessed 1,285 patients' clinical data from the ICU of the Affiliated Huaian No. 1 People's Hospital of Nanjing Medical University, from January 1, 2016, to December 31, 2019. Patients who met the sepsis-induced AKI criteria (171 cases) were further analyzed and compared. In the present study, we found that abdominal abscess and ileocecal and appendiceal disease were the etiologies with higher mortality in sepsis-induced AKI patients and that the values of PCT, PCT/Alb, CRP, and CRP/Alb were significantly higher in the non-survival group compared to the survival group. Further analysis discovered that both PCT and PCT/Alb were independent risk factors for poor prognosis in patients with sepsis-induced AKI and that PCT/Alb exhibited a more robust risk assessment value.

Inflammation and malnutrition are highly associated with the pathogenesis of AKI (12–14). CRP, an acute-phase protein synthesized by the liver, and PCT, a marker for detecting infection/inflammation, have been receiving attention in predicting mortality of diseases, including AKI (15–18). CRP was implicated in renal fibrosis and renal ischemia-reperfusion injury, and its increase was significantly related to the occurrence and mortality of AKI (19). Gaini et al. revealed that elevation of serum CRP concentration was closely associated with the mortality of patients in the setting of sepsis and critical illness (16). Additionally, a high level of PCT was a risk predictor for patients with sepsis-induced organ dysfunction (including kidney damage) as well as AKI, and it also negatively correlated with patients' prognosis (20–23). Moreover, serum albumin, prealbumin, and cholesterol are markers recommended by the International Society of Renal Nutrition and Metabolism (ISRNM) to assess the nutritional status of patients (24). Malnutrition (such as lower concentration of prealbumin and cholesterol) was a risk predictor for patients with severe trauma and AKI (25). Accordingly, in the present study, we found higher levels of CRP and PCT and a slight (non-significant) decrease tendency in respect to Alb concentration in the non-survival group when compared to the survival group, suggesting that CRP, PCT, and Alb might also play pivotal roles in sepsis-induced AKI.

In pathological conditions, the interactions between inflammation and malnutrition are intimate and complicated. For example, inflammation could result in malnutrition, while malnutrition, in turn, served as a detrimental factor for the management of inflammation. In this context, a single marker (inflammation or malnutrition) can hardly provide a robust risk prediction for diseases, such as AKI. Indeed, some studies utilized the integration marker to predict the risk of diseases. For instance, Pinilla et al. reported that the value of CRP/prealbumin was correlated to the severity of organ dysfunction in critically ill patients (26). Besides, a high ratio of CRP/Alb indicated higher inflammation superimposed with malnutrition status and was inversely associated with the prognosis of patients with acute myocardial infarction (27, 28). Notably, Xie et al. discovered that the CRP/prealbumin ratio could predict the risk of mortality in patients with hospital-acquired AKI (29). However, no research about whether CRP/Alb and PCT/Alb can evaluate the prognosis of patients with sepsis-induced AKI has been reported so far. Herein, we found that the values of PCT, PCT/Alb, CRP, and CRP/Alb were significantly higher in the non-survival group and that PCT, PCT/Alb were independent risk factors for poor prognosis in patients with sepsis-induced AKI. As expected, the combination markers (PCT/Alb) exhibited more predictive value than either single marker in sepsis-induced AKI patients. Intriguingly, it seems that the PCT value might contribute to the principal proportion of the predictive value of the PCT/Alb due to its significant change between the survival group and non-survival group, while the Alb value can enhance the predictive power of PCT even though a slight (non-significant) reduction in the non-survival group, implying that the non-significantly changed nutrition markers in sepsis-induced AKI patients also have crucial predictive value, especially when combined with inflammation markers.

Overall, we found a robust predictor (PCT/Alb) of mortality in sepsis-induced AKI patients through a long-time retrospectively study, indicating, at least partly, that when higher levels of PCT/Alb appeared in patients with sepsis-induced AKI, a poorer prognosis and more aggressive diagnostic and therapeutic interventions were needed to avoid mortality. However, some limitations still existed in our study: (i) this was an observational, single-center study with relatively small cohort size; (ii) apart from Alb, other nutritional markers and variables (such as prealbumin, cholesterol, and MUAC) should also be taken into consideration when combined with inflammatory markers; and (iii) the involved population was composed of heterogeneous AKI patients in a tertiary comprehensive hospital and a potential selection bias might influence the result. Hence, a multi-center study with adequate cohort size and a comprehensive assessment of the combined diagnosis value of nutrition and inflammation should be performed in the following study to further confirm the predictive value of PCT/Alb in the poor prognosis of patients with sepsis-induced AKI and to find other potentially valuable combination markers.

## Conclusions

In conclusion, the present study first evaluated the correlation between CRP/Alb and PCT/Alb levels and the mortality of sepsis-induced AKI patients. Higher PCT/Alb level was strongly associated with higher mortality in sepsis-induced AKI patients. Therefore, it was a robust predictor marker of mortality in these patients.

## Data Availability Statement

The original contributions presented in the study are included in the article/Supplementary Material, further inquiries can be directed to the corresponding author/s.

## Ethics Statement

This study was conducted following the Declaration of Helsinki and was approved by the Ethical Committee of the Affiliated Huaian No. 1 People's Hospital of Nanjing Medical University. All patient data were analyzed in anonymity. Patient consent was waived by the ethics committee, as no individual data were published, nor was any intervention performed on patients.

## Author Contributions

LC summarized the AKI factors, wrote and edit the manuscript. HZ designed the content, reviewed and edited the manuscript, others summarized the content of the abdominal infection-evoked sepsis. All authors listed have made a substantial, direct and intellectual contribution to the work, and approved it for publication.

## Conflict of Interest

The authors declare that the research was conducted in the absence of any commercial or financial relationships that could be construed as a potential conflict of interest.
